# D- and N-Methyl Amino Acids for Modulating the Therapeutic Properties of Antimicrobial Peptides and Lipopeptides

**DOI:** 10.3390/antibiotics12050821

**Published:** 2023-04-27

**Authors:** Maria Veronica Humpola, Roque Spinelli, Melina Erben, Virginia Perdomo, Georgina Guadalupe Tonarelli, Fernando Albericio, Alvaro Sebastian Siano

**Affiliations:** 1 Laboratorio de Péptidos Bioactivos, Departamento de Química Orgánica, Facultad de Bioquímica y Ciencias Biológicas, Universidad Nacional del Litoral, Santa Fe S3000ZAA, Argentina; mvhumpola@fbcb.unl.edu.ar (M.V.H.); roquespinelli@gmail.com (R.S.); erbenmelina@gmail.com (M.E.); tonarelli@fbcb.unl.edu.ar (G.G.T.); 2 Consejo Nacional de Investigaciones Científicas y Técnicas (CONICET), Buenos Aires C1425FQB, Argentina; vperdomo@fbioyf.unr.edu.ar; 3 Área Parasitología, Facultad de Ciencias Bioquímicas y Farmacéuticas, Universidad Nacional de Rosario, Rosario S2002KTT, Argentina; 4 School of Chemistry and Physics, University of KwaZulu-Natal, Durban 4001, South Africa; 5 Consorcio Centro de Investigación Biomédica en Red de Bioingeniería, Biomateriales y Nanomedicina (CIBER-BBN), Networking Centre on Bioengineering, Biomaterials and Nanomedicine, Department of Organic Chemistry, University of Barcelona, 08028 Barcelona, Spain

**Keywords:** antimicrobial peptides, N-methyl amino acids, D-amino acids, enzymatic stability, toxicity

## Abstract

Here we designed and synthesized analogs of two antimicrobial peptides, namely C10:0-A2, a lipopeptide, and TA4, a cationic α-helical amphipathic peptide, and used non-proteinogenic amino acids to improve their therapeutic properties. The physicochemical properties of these analogs were analyzed, including their retention time, hydrophobicity, and critical micelle concentration, as well as their antimicrobial activity against gram-positive and gram-negative bacteria and yeast. Our results showed that substitution with D- and N-methyl amino acids could be a useful strategy to modulate the therapeutic properties of antimicrobial peptides and lipopeptides, including enhancing stability against enzymatic degradation. The study provides insights into the design and optimization of antimicrobial peptides to achieve improved stability and therapeutic efficacy. TA4(dK), C10:0-A2(6-NMeLys), and C10:0-A2(9-NMeLys) were identified as the most promising molecules for further studies.

## 1. Introduction

Antimicrobial peptides (AMPs) are a class of small molecules produced by numerous living organisms as part of their host innate immune response to infection [1,2,3]. AMPs derived from insects and other species have demonstrated the ability to eliminate invading pathogens, holding promise with regard to the prospect of developing AMPs as alternatives to antibiotics [4,5]. AMP research endeavors started back in the 1980s with the discovery of insect cecropins by Hans Boman, human α-defensins by Robert Lehrer, and magainins by Michael Zasloff [6]. Over 3300 kinds of AMPs have now been found in a vast number of biological sources, ranging from microbes to plants and animals [7]. These peptides may have optimal properties for further drug development. In this regard, they can permeabilize and disrupt the bacterial membrane, regulate the immune system, exert broad-spectrum antibiofilm activity, and show a reduced propensity for selective bacterial resistance [8,9,10,11]. Although the use of AMPs has primarily been limited to topical infections due to their relatively narrow druggability and lack of special unique protocols to assess pharmacodynamics, the advantages of these molecules over conventional antibiotics have attracted wide attention from the research community [12,13].

Some AMPs have already found clinical and commercial applications [14]. However, the future design and optimization of novel AMPs call for efforts to improve the antimicrobial activity of these molecules, minimize their cytotoxicity, and reduce their proteolytic degradation or biological fluid inhibition [15,16,17,18]. In this regard, the goal is to design new molecules with high specificity for prokaryotic cell membranes but minimal toxicity toward eukaryotic membranes. To pursue this objective, methodologies of combinatorial chemistry and/or rational design can be used. The sequences of these new molecules may contain non-proteinogenic amino acids, such as D-amino acids, β-amino acids, and N-methyl amino acids, or their conformational structure can be modified by cyclization to improve stability against proteases and interaction with the membranes of microorganisms [17,18,19,20,21]. Here, we report the optimization of novel antimicrobials by the addition of D- and N-methyl amino acids to not only improve the biological activity of the molecules but also their therapeutic properties.

## 2. Results and Discussion

In a previous study, we reported a potent antimicrobial lipopeptide, namely C10:0-A2, and a cationic decapeptide (IKQVKKLFKK) which was conjugated with decanoic acid (C10:0) [22]. This lipopeptide exhibited potent broad-spectrum activity against gram-positive and gram-negative bacteria with a MIC range of 1.4 to 2.8 μM, and was able to inhibit the degrowth of yeast (MIC = 90.6 μM). Nevertheless, its selectivity is low due to its moderate hemolytic activity, around 50% of hemolysis at 200 μM. We demonstrated that C10:0-A2 adopted an amphipathic α-helix structure in bacterial membrane mimetic vesicles but remained unstructured in the presence of eukaryotic membrane mimetic vesicles. Therefore, we suggest that the adoption of a stable secondary structure is a key factor for antibacterial activity, while the hemolytic activity is governed by the lipopeptide hydrophobicity. In a later study, we went on to describe the design and synthesis of a cationic 12 residue α-helical amphipathic peptide, TA4, based on a pharmacophore motif of bacteriocin Pln 149 (KLFK), which resulted in a promising antimicrobial with a MIC range of 2.5 to 10.2 μM against bacteria, and 40.8 μM against yeast. [23]. This peptide showed moderate hemolytic activity (around 40% of hemolysis at 200 μM), therefore affecting their selectivity and therapeutic window. Structurally, TA4 has high cationicity (+7) which comprises 50% of hydrophobic amino acids, and adopts an amphipathic α-helical secondary structure in the presence of DPPG vesicles that mimic bacterial membranes, resulting in an optimal combination to ensure membrane permeabilization. Due to the antimicrobial properties and structural features described above, these molecules are of great potential as possible candidates for the development of alternative antimicrobials. It is widely known that natural peptides are susceptible to degradation by digestive and serum enzymes, which reduce the oral bioavailability and half-life circulation of these molecules, and consequently, diminishes their therapeutic efficacy [15]. To improve the therapeutic properties of the two aforementioned peptides, we designed and synthesized analogs in which non-proteinogenic amino acids, namely D- and N-Me, were introduced.

### 2.1. Peptide Synthesis and Physicochemical Characterization

The synthetic peptide and lipopeptides containing D- and N-methyl amino acids are shown in Table 1. All the compounds were synthesized as amide peptides by solid-phase peptide synthesis (SPPS).

HPLC analyses showed that the substitution with D- and N-methyl amino acids decreased the retention time (rt) (see Table 1), and thus, their experimental hydrophobicity. Therefore, this observation indicates that the substitution affects the interaction of the peptides and lipopeptides with the stationary phase of the column, possibly because the peptide sequence adopts a distinct conformation in a hydrophobic environment. Several studies report that the substitution by D- or N-methyl amino acids affects the adoption of the secondary structure of helical peptides, which causes a decrease in the retention time when the compounds elute from HPLC columns [24,25]. Using HPLC analysis, De Vleeschouwer et al. (2017) observed that the retention time of N-methylated analogs of a cyclic lipopeptide (pseudodesmin A) significantly decreased, as did their hydrophobicity. Although N-methylation is expected to increase hydrophobicity, and thus retention time, these authors found that when N-methylation occurs at the center position of the peptide sequence, as demonstrated by 1HNMR analyses, there was a dramatic impact on the overall conformation of the compound. In addition, the presence of an N-alkyl group in an amino acid within the peptide sequence eliminates the possibility of inter- and intra-molecular hydrogen bond formation, thus destabilizing or modifying the secondary structures that can be adopted by the peptide [26].

On the other hand, acylation confers that the peptide has capacity to self-assemble into nanostructures, resulting in a surfactant-like structure, which may affect the extent of peptide insertion and disruption of bacterial membrane integrity. In this work, the CMC of the lipopeptides was determined by conductimetry in Milli-Q water. All the lipopeptides adopted a micellar assembly with CMC values ranging from 2.0 to 5.7 mM (Table 1). The CMC values of all the lipopeptides containing N-methyl amino acids were lower than those of C10:0-A2, thereby indicating that the presence of N-methyl amino acids in the peptidyl motif facilitated self-assembly into micelles. This effect appeared to be more important when substitution by an N-methyl amino acid was performed on amino acids located on the non-polar face of the amphipathic helix (CMC values of C10:0-A2(8-NMePhe) = 2.01 mM). For all the lipopeptides, the CMC values remained above the MIC values, which could indicate that these molecules interact with the cell membrane as monomers and, consequently, that this property would not be necessary for antimicrobial activity. This notion is in concordance with reports in the literature on short cationic lipopeptides [27,28].

### 2.2. Antimicrobial Activity

Table 2 shows the antimicrobial activity of the peptides and lipopeptides against bacterial strains and yeast. The substitution of natural amino acids by N-methyl amino acids allowed us to obtain analogs with similar antibacterial activity, and in some cases, greater activity than non-substituted molecules, particularly against *P. aeruginosa* strain ATCC 27853. Only some analogs showed a slightly higher MIC value than the non-substituted ones against *E. faecalis*. For the lipopeptides, the substitution of L-Lys by L-NMe-Lys in positions six and nine was the most favorable for antibacterial activity, whereas in analogs with a replacement of L-Phe by L-NMe-Phe and L-Lys by L-NMe-Lys in positions eight and five, respectively, a slight reduction of activity was observed. Thus, this finding suggests that these amino acids are important for the biological activity of the compounds. For peptide TA4, the substitution of two L-Phe by L-NMe-Phe was less favorable against gram (+) bacterial strains.

In a structure–activity relationship study, Velkov et al. addressed the effect of N-methylation at different positions (two to seven) of the Leu10-teixobactin depsipeptide. In general, they observed that N-methylation had a negative effect on antimicrobial activity. Those authors suggested that this loss of activity was due to a conformational change and/or a reduction in the ability to form a multimeric active structure by blocking hydrogen bond formation [29].

In contrast, we found that the substitution by D-amino acids was less suitable for antibacterial activity. TA4(dK) showed satisfactory inhibitory activity against all the bacterial strains tested (MIC values = 5 to 20.4 µM), although the MIC values were higher than those of TA4, while C10:0-A2(dk) retained the inhibitory activity only against gram (−) bacterial strains. These results are consistent with those reported by Zhao et al. (2016) for a D-Lys substituted analog of polybia-MPI: an α-helical cationic peptide isolated from the wasp *Polybia paulista* (IDWKKLLDAAKQIL-NH2). The replacement of three L-Lys by D-Lys resulted in a loss of antimicrobial activity against gram (+) and (−) bacteria. The authors suggested that this effect on antibacterial activity was due to a reduction in α-helical conformation in a membrane-mimicking environment [21].

Concerning antifungal activity, most of the substituted analogs inhibited the growth of the two yeast strains tested. In particular, C10:0-A2(6-NMeLys) showed improved inhibitory activity against *C. albicans*. For TA4, the substitution of the seven L-Lys by D-Lys considerably reduced inhibitory activity against the two yeast strains.

Most of the peptide and lipopeptide analogs synthesized in this work showed antibacterial (0.7–40 μM) and antifungal (44.9–181.2 μM) activity similar to that of antimicrobial peptides that are in clinical trials for FDA approval. Pexiganan, a lysine-substituted analog of magainin-2 is a potent broad-spectrum antibacterial (MIC values: 2–64 μg/mL (0.8–26 μM). It is being developed as a topical therapy for infected foot ulcers in patients with diabetes [30] Likewise, omiganan, a peptide comprising 12 amino acid residues that is derivate of indolicin (ILRWPWWPWRRK-amide) with a broad-spectrum of antimicrobial activity (MIC values against gram (+) and (−) bacteria: 0.5–256 ug/mL (0.25–130.5 uM) and against *Candida* sp.: 16 to 256 ug/mL (8–130.5 uM) is being developed as a topical antimicrobial agent [31,32].

### 2.3. Toxicity Characterization and Therapeutic Index (TI)

In the development of new drugs for human health, low toxicity towards host cells is a desirable property. In this regard, the assessment of toxicity is an important issue. Here, we evaluated the hemolytic and cytotoxic activity of the new synthetic compounds in vitro.

**Figure 1:**
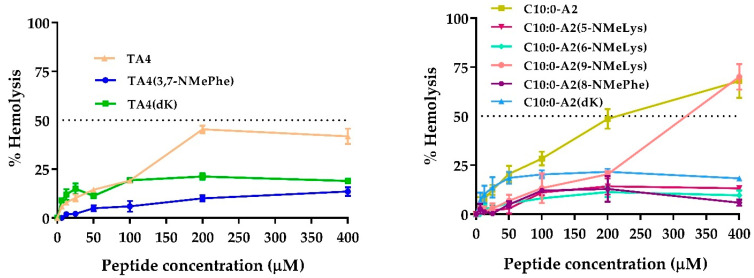
Hemolytic activity of the synthetic peptides (**left**) and lipopeptides (**right**). Curve doses vs. response: % of hemolysis as a function of peptide concentration.

Hemolytic activity was determined using red blood cells and the hemoglobin released was measured at 405 nm. The percentage of hemolysis corresponding to the synthetic compounds is shown in Figure 1 while the HC_50_ values are provided in Table 3. TA4 and C10:0-A2 showed moderate hemolysis at higher concentrations (400 μM), achieving around 65% and 45% of hemolysis, respectively. Substitutions by N-methyl and D-amino acids reduced the hemolytic activity of the peptides and lipopeptides. Most of the substituted analogs showed less than 20% of hemolysis at all the concentration ranges tested, except C10:0-A2(9-NMeLys), which at higher concentrations presented a similar hemolysis profile to that of C10:0-A2. For all the peptides and most of the lipopeptides, the HC_50_ values exceeded 400 μM. Indeed, the HC_50_ values were lower only for C10:0-A2(9-NMeLys) and C10:0-A2, which were around 300 and 200 μM, respectively.

Consistent with these results, Li et al. (2013) [33] demonstrated that the substitution of the residues involved in the formation of internal hydrogen bonds of gramicidin S, a β-type structure peptide, by N-methyl amino acids caused a significant decrease in the hemolytic activity of the analogs. In contrast, the substitution of residues involved in the formation of external hydrogen bonds did not affect this parameter. The authors suggested that the latter observation could be attributed to the alteration of the secondary structure and the change in amphipathicity.

Many studies have demonstrated that high amphipathicity, high hydrophobicity, and a high tendency to adopt secondary structure are key parameters responsible for high toxicity to mammalian cells [34,35,36].

**Figure 2:**
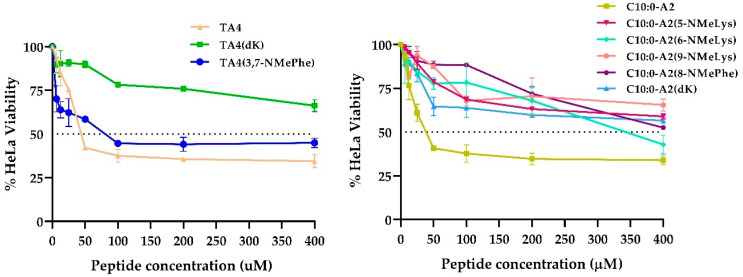
Cytotoxicity of synthetic peptides (**left**) and lipopeptides (**right**). Curve doses vs. response: % of HeLa cell line viability as a function of peptide concentration.

We assessed the cytotoxicity of the compounds synthesized using HeLa cells. The viability percentage of synthetic compounds is shown in Figure 2. The non-substituted compounds, namely TA4 and C10:0-A2, presented significant toxicity, reducing HeLa viability by more than 60%. In contrast, the vast majority of substituted analogs were less cytotoxic at all the concentrations tested (>50% of HeLa viability). The IC_50_ values calculated for each compound are shown in Table 3. For all substituted analogs, the IC_50_ value was equal to or higher than 400 μM, and were considered non-cytotoxic, except for TA4(3,7-NMePhe), which showed an IC_50_ value of around 86 μM, even though it was less cytotoxic than TA4 (IC_50_ = 46 μM).

It is important to note none of the compounds showed cytotoxicity or hemolytic activity in the MIC concentration range.

For most of the analogs, hemolytic activity and cytotoxicity had correlated, thereby suggesting that the modifications made were effective against both eukaryotic cells. Exceptions were the peptide TA4(3,7-NMePhe), which exhibited moderate cytotoxicity (IC_50_: 86% μM) but was not hemolytic (HC_50_ > 400 μM), and the lipopeptide C10:0-A2(9-NMeLys), which was moderately hemolytic (HC_50_: 300 μM) but not cytotoxic (IC_50_ > 400 μM).

The selectivity of a compound can be assessed using the therapeutic index (TI). This parameter can be increased by enhancing antimicrobial activity, decreasing hemolytic activity, or by a combination of both. In this work, the TI was calculated as the relationship between the HC_50_ or IC_50_ values and mean MIC against different microorganisms (yeast and gram (+) and (−) bacteria) (Table 3). For peptides, TA4(dK) presented the best TI against all types of microorganisms. Although the antimicrobial activity of this compound was lower than that of the unsubstituted peptide, the TI was higher due to its lower toxicity against red blood cells and HeLa cells. TA4(3,7-NMePhe), on the other hand, was able to increase TI values only against gram (−) bacteria compared with TI values of TA4. However, for lipopeptides, C10:0-A2(9-NMeLys) and C10:0-A2(6-NMeLys) showed higher TI values than C10:0-A2, especially C10:0-A2(6-NMeLys) against gram (−) bacteria. These results are explained by their lower toxicity compared to that of C10:0-A2.

### 2.4. Enzymatic Stability Characterization in the Presence of Digestive Enzymes and Serum Proteases

**Figure 3:**
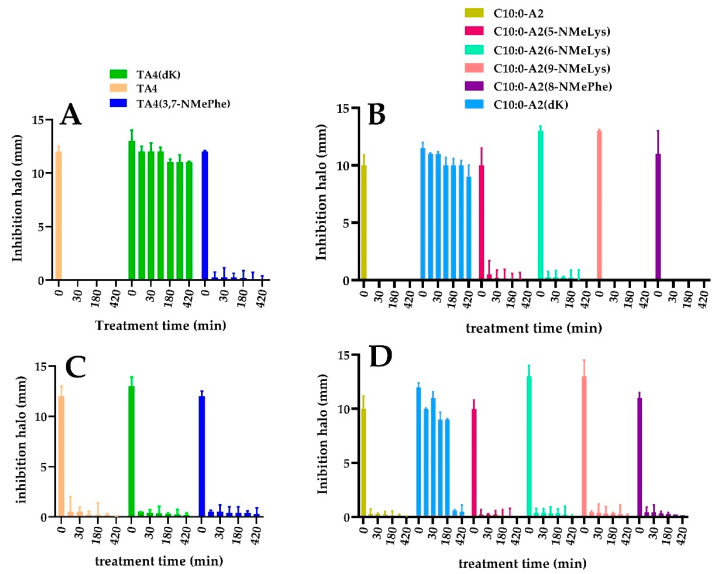
Enzymatic stability characterization in the presence of trypsin (**A**—peptides; **B**—lipopeptides) and quimotripsyn (**C**—peptides; **D**—lipopeptides). Curve doses vs. response: inhibition halo (mm) as a function of treatment time (min).

The enzymatic stability of the peptides and lipopeptides in the presence of trypsin and chymotrypsin was evaluated in vitro by measuring residual antimicrobial activity. These results are shown in Figure 3.

Substitution of L-Lys for D-Lys in C10:0-A2 and TA4 resulted in increased stability against trypsin degradation, retaining 80% and 85% inhibition activity against *S. aureus* strain ATCC 25923 after 7 h of treatment, respectively. These results indicate that the replacement of all sites susceptible to trypsin cleavage by non-proteinogenic D-series amino acids, markedly increased the stability of the compounds. In the presence of chymotrypsin, these two peptides were less stable compared to trypsin. C10:0-A2(dK) retained 75% of its antimicrobial activity after 3 h of treatment. However, a total loss of activity was observed after this time. In contrast, TA4(dK) showed a full loss of activity after 30 min of treatment. Multiple substitutions by D-Lys in C10:0-A2 seemed to have a favorable effect on stability against chymotrypsin. This observation might be explained by a conformational change that makes the target site less accessible, thus requiring enzyme activity for longer. This was not observed for TA4(dK), possibly because its sequence holds more sites susceptible to chymotrypsin action. On the other hand, the replacement of one or two proteinogenic amino acids by N-methyl amino acids in TA4 and C10:0-A2 was not enough to improve enzymatic stability.

**Figure 4:**
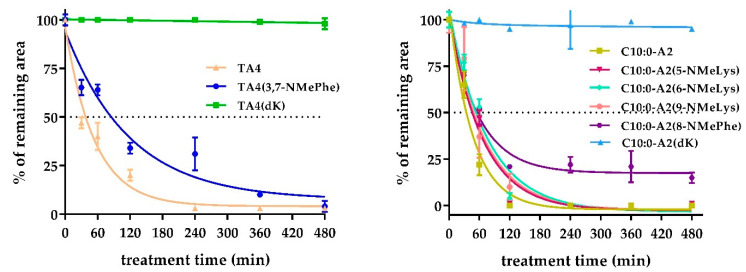
Enzymatic stability characterization in the presence of serum protease of peptides (**left**) and lipopeptides (**right**). Curve doses vs. response: % of remaining area as a function of treatment time (min).

Peptide stability in human serum was evaluated by HPLC analyses. The remaining area versus treatment time was plotted (Figure 4).

Both TA4 and C10:A2 were degraded by serum proteases with about 40% and 20% of the remaining area after 1 h of incubation, respectively. Substitution of L-Lys by D-Lys enhanced the serum stability of TA4 and C10:0-A2, with 100% of the remaining area after 8 h of incubation.

On the other hand, substitution with N-methyl amino acids also enhanced stability in serum, increasing from 40% to 65% of the remaining area of peptides and from 20% to 50% for lipopeptide analogs after 1 h of exposure. Although both strategies improved enzymatic stability to serum protease, multiple substitutions with non-proteinogenic acids had a more significant effect on this property than single substitutions.

These results are in concordance with those reported by Dong et al. (2012), where at selected sites of DhHP-6, tri-N-methylation (a deuterohemin-mimetic peptide conjugate of microperoxidase), showed higher resistance to serum and digestive proteolytic degradation and higher apparent permeability coefficient than mono- and di-substituted analogs [37].

Hong et al. (1999) studied the effect of D-amino acid substitution in the KKVVVFKVKVKFKK peptide on enzyme stability in serum. These authors demonstrated that the substitution of susceptible sites increased the enzymatic stability of the analogs and that this increase in stability correlated with an increase in the number of susceptible sites blocked simultaneously [38].

In another study, Gao and coworkers reported similar results in a phylloseptin-PT analog: PS-PT2. Here the substitution of five L-Lys by D-lys was more effective at improving stability against trypsin and serum proteases than the individual L-Lys by D-Lys substitution in position seven [39].

### 2.5. Secondary Structure Determination by Circular Dichroism (CD)

**Figure 5:**
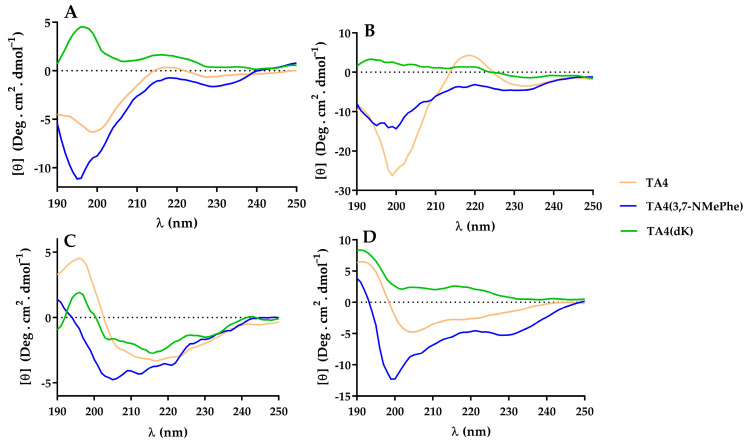
Circular Dichroism spectra of peptides in (**A**) water; (**B**) DPPC vesicles; (**C**) DPPG vesicles; and (**D**) TFE/water (50%, *v*/*v*). Peptide concentration: 0.2 mg/mL.

The CD spectra of the substitution analogs of TA4 in water and DPPC (Figure 5A,B) showed that all the analogs were predominantly unstructured.

The analyses of the CD spectra of TA4(dk) and its deconvolution by the Selcon3 and Contin methods showed that the presence of TFE:H_2_O (50% *v*/*v*) presented a greater contribution of beta structure and turn (45%), with 28% of the unordered structure (see Figure S1). On the other hand, in the presence of DPPG, the peptide was less ordered. In contrast, TA4 showed greater contributions of the alpha helix in TFE (45%) and DPPG (36%), and also its structure was more stable in TFE (only 24% of unordered structure). These results revealed that the incorporation of D-amino acids in the TA4 sequence caused a significant modification to its secondary structure.

The deconvolution of the CD spectra of the analog TA4(3,7-NMePhe) showed that the peptide was partially structured (with 32% of unordered structure) in the presence of DPPG. In comparison with TA4, the presence of N-MePhe caused a conformational change in the original structure of the TA4 analog, with a slight increase in the beta and turn structure (from 27 to 33%), and a reduction of the helical structure (from 36% to 27%). Similar results were found with TFE:H_2_O 50%.

**Figure 6:**
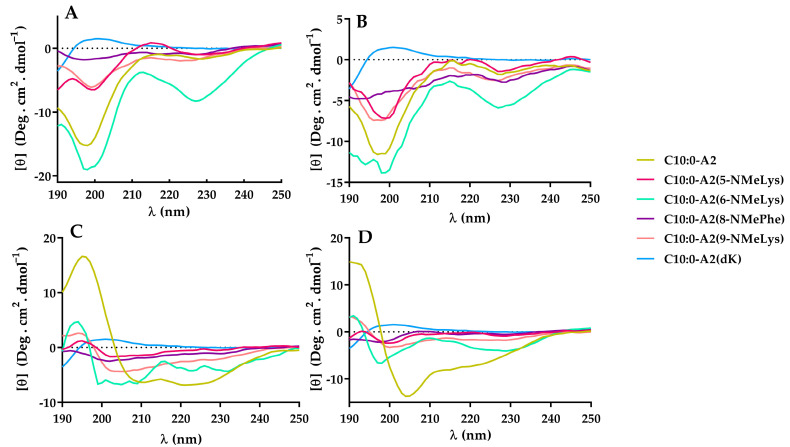
Circular Dichroism spectra of lipopeptides in (**A**) water; (**B**) DPPC vesicles; (**C**) DPPG vesicles; and (**D**) TFE/water (50%, *v*/*v*). Peptide concentration: 0.2 mg/mL.

The CD spectra of all C10:0-A2 lipopeptide analogs in water showed that they were predominantly unordered, which is consistent with the presence of a minimum at 198 nm (or maximum in cases of C10:0-A2(dK)), except for C10:0-A2(6-NMeLys) (Figure 6A), whose deconvolution showed 26% of α-helix and 16% of turn contribution (see Figure S2), making this the most structured analog in water (only 44% of unordered structure).

In the presence of TFE and DPPG, the analogs that contained NMeLys or D-Lys were less structured than C10:0-A2 (Figure 6C,D). This result could explain the fact that most of the analogs showed less antimicrobial activity than C10:0-A2. This observation would then suggest that N-Methyl amino acids and D-amino acids affect the secondary structure.

On the basis of the spectra analyses and deconvolution in DPPG, analog C10:0-A2(6-NMeLys)—being the most ordered one (only 30% of unordered structure)—emerged as the one with the highest α-helix contribution (48%). C10:0-A2(9-NMeLys) showed similar characteristics.

On the other hand, the spectra of C10:0-A2(5-NMeLys) and C10:0-A2(8-NmePhe) showed the contribution of beta and turn structure (24–30%) and more than 50% of unordered structure.

These results reveal that the incorporation of N-Methyl amino acids in the C-terminus and the middle part of the A2 sequence disturbed the secondary structure. The analogs with non-proteinogenic amino acid substitutions in positions six and nine showed higher structuration and also presented greater antimicrobial activity than those substituted in positions five and eight. These observations suggest that, for this lipopeptide, the adoption of an alpha helix structure is a key feature for antimicrobial activity.

Finally, in the presence of DPPC, all the analogs studied were predominantly unordered (Figure 6B); an observation that is consistent with their low hemolytic activity.

## 3. Material and Methods

### 3.1. Peptide Synthesis

All the compounds in this study were obtained as C-terminal amides by Fmoc solid-phase peptide synthesis (SPPS) (Table 1). To prepare the lipopeptides, different fatty acid chains were added on the N-terminus of peptide bound to the resin using standard protocols. For the coupling reactions, (1H-benzotriazol-1-yl)(dimethylamino)-methylene]-N-methylmethanaminium tetrafluoroborate N-oxide (TBTU) and diisopropylethylamine (DIEA) were used. Couplings of N-methyl amino acids and the subsequent amino acid were performed by benzotriazol-1-yl-oxytripyrrolidinophosphonium hexafluorophosphate (PyBOP) and DIEA. Fmoc was removed with 20% piperidine in N, N-dimethylformamide (DMF) (*v*/*v*). Final cleavage from the resin was achieved by a mixture of trifluoroacetic acid (TFA)/H_2_O/triisopropyl silane (TIS) (95:2.5:2.5) (*v*/*v*). The crude peptides were precipitated in dry cold diethyl ether, centrifuged, and washed several times with cold diethyl ether until scavengers were removed. The products were then dissolved in water and lyophilized twice.

Peptide and lipopeptides were analyzed by RP-HPLC (Waters) using an Atlantis (Waters) C18 analytical column (5 μm, 4.6 mm × 150 mm) for peptides and a Jupiter (Phenomenex) C4 (5 μm, 300 Å, 250 × 4.60 mm) analytical column for lipopeptides. For elution purposes, a linear gradient from 15% to 60% of acetonitrile (ACN) in H_2_O containing 0.1% TFA, at a flow rate of 0.8 mL/min was used for lipopeptides, and a linear gradient from 5% to 80% of acetonitrile with 0.1% TFA at a flow rate of 0.8 mL/min for peptides. Absorbance was measured at 220 nm. The crude products synthesized were purified by HPLC using a semi-preparative reverse-phase (RP) C18 column (Jupiter-Proteo Phenomenex, 10 µm, 90 Å, 250 × 10 mm), and Mass spectrometric data were obtained using a MALDI-TOF-TOF spectrometer, Ultraflex II (Bruker), in the Mass Spectrometry Facility CEQUIBIEM, Argentina.

### 3.2. Antimicrobial Activity

#### 3.2.1. Minimal Inhibitory Concentration (MIC) against Bacteria

The MIC against bacterial strains was determined by the modified broth microtiter dilution method, following the procedures proposed by R.E.W. Hancock Laboratory for testing antimicrobial peptides [40]. The target strains *Escherichia coli* ATCC 35218, *Pseudomonas aeruginosa* ATCC 27853, *Enterococcus faecalis* ATCC 29212, and *Staphylococcus aureus* ATCC 25923 belong to the American Type Culture Collection (ATCC). The Methicillin-Resistant *Staphylococcus aureus* BSF FBCB1313 strain (MRSA) was provided by the Clinical Bacteriology Section of FBCB-UNL. All strains were activated by culture for 24 h at 37 °C in Mueller-Hinton Broth (MHB) (Biokar Diagnostics, Cedex, France). Each inoculum was taken and adjusted to a cellular concentration of 5 × 10^5^ colony-forming units (CFU)/mL in diluted MHB. All the peptides were dissolved in bovine serum albumin buffer with the addition of 0.01% acetic acid; 100 µL of each inoculum was added to 11 µL of peptide solution in serial 2-fold dilutions and were incubated for 18–24 h at 37 °C. The MIC was the lowest peptide concentration that inhibited visible growth of each bacterial strain. The test was conducted in triplicate.

#### 3.2.2. Minimal Inhibitory Concentration (MIC) against Yeast

The MIC against yeast strains was determined by the broth microtiter dilution method following the conditions of NCCLS document M27-A. The target strains *Candida albicans* DBFIQ CA 1, *C. albicans* PEEC 2, and *Candida tropicalis* DBFIQ 3, all belonging to the Culture Collection of Microbiology and Biotechnology Sections-FIQ-UNL, were activated by culture for 24 h at 30 °C in Sabouraud Dextrose Agar (SDA) (Biokar Diagnostics, Cedex, France). Each inoculum was taken and the cellular concentration was adjusted to 2 × 10^3^ CFU/mL in Sabouraud Dextrose Broth (SDB) (Biokar Diagnostics, Cedex, France). Next, 50 µL of these inocula was added to 50 µL of peptide solution in serial 2-fold dilutions. The plates were incubated for 48 h at 30 °C. The MIC was considered the lowest peptide concentration that inhibited visible growth of each yeast strain. The test was conducted in triplicate.

### 3.3. Toxicity Characterization

#### 3.3.1. Hemolysis Assay

Erythrocyte lysis was determined using the following previously optimized protocols [41]. Human erythrocytes from a healthy voluntary donor were isolated by centrifugation (3000 rpm for 10 min) after washing three times with Physiological Solution (PS). Erythrocyte solutions were prepared at a concentration of 0.4% (*v*/*v*) in PS. Test tubes containing 200 µL of erythrocyte solution were incubated at 37 °C for 60 min with 200 µL of peptide solution at concentrations ranging from 6.25 to 400 µM. After centrifugation at 3000 rpm for 5 min, supernatant absorbance was measured at 405 nm. Lysis induced by 1% Triton X-100 was taken as a 100% reference value.

#### 3.3.2. Cytotoxicity Assay

The 3–4,5-dimethylthiazol-2,5-biphenyl tetrazolium bromide (MTT) cell viability assay was used to assess the cytotoxic activity of synthetic compounds against the human HeLa line. HeLa cells were seeded in 96-well plates at 1 × 10^5^ cells/well, in which the peptide solution was added at different concentrations (3.125–400 μM) for 24 h of incubation. Next, MTT reagent (Sigma-Aldrich, St. Louis, MO, USA) was added, and the cells were incubated for 2 h. Then, 100 μL of dimethylsulfoxide (DMSO) was added to each well to dissolve formazan crystals, and the plates were read at 595 nm. Finally, the IC_50_ values of the peptides and lipopeptides were calculated as the mean of the concentrations at which each compound caused a 50% decrease in cell viability in two independent experiments, each with three replicates.

### 3.4. Calculation of the Therapeutic Index (TI)

The TI is defined as the relationship between the concentration that caused 50% of hemolysis (HC_50_) or a 50% reduction in HeLa cell viability and the MIC. Thus, higher TI values indicate greater antimicrobial specificity. When a peptide did not surpass 50% of hemolysis (or reduction of viability) at any of the concentrations tested, a value of 400 µM was used to calculate the TI. The average MIC for each peptide against different microorganism groups was used to calculate the TI value.

### 3.5. Characterization of Enzymatic Stability

#### 3.5.1. Peptide Stability in the Presence of Digestive Enzymes

500 µL of a trypsin or chymotrypsin solution in ammonium bicarbonate buffer (0.03 M pH = 7.9) at a concentration of 2 mg/mL was added to 500 µL of peptide solution (concentration: 2 mg/mL). The mixtures were incubated for 7 h at 37 °C. Aliquots were taken at various time points (0, 15, 30, 60, 180, 300, and 420 min). Subsequently, the enzyme reaction was immediately stopped by thermal shock (80 °C for 10 min). All fractions collected were lyophilized twice.

The enzymatic stability of the peptides and lipopeptides was determined by measuring residual antimicrobial activity using the agar diffusion method [42]. To this end, 60 µL of the peptide (treated and untreated) in phosphate buffer (0.1 mM pH: 5.5) was added to a 7 mm well on an agar plate previously seeded with 1 mL of a fresh culture of *S. aureus* ATCC 25923. The plates were incubated for between 18 and 24 h at 37 °C, and then the inhibition halo diameter was measured. The test was conducted in duplicate.

#### 3.5.2. Peptide stability in Serum

A 50% (*v*/*v*) human serum solution in phosphate buffer (pH: 7.2) was added to the peptide solution (concentration: 2 mg/mL) and incubated for 8 h at 37 °C. Aliquots were taken at different time points (0, 30, 60, 120, 240, 360, and 480 min) and serum proteins were immediately precipitated with a mixture of ACN-water-TFA (89:10:1). They were kept at 4 °C for 45 min and then centrifuged for 15 min at 10,000 rpm. The supernatants were then analyzed by HPLC-RP using a C18 analytical column (Beckman, Indianapolis, IN, USA). A linear gradient from 5% to 50% of ACN in H_2_O containing 0.1% TFA was used, at a flow rate of 0.8 mL/min. Absorbance was measured at 220 nm. The result was expressed as the percentage of area remaining vs. treatment time. The test was conducted in duplicate.

### 3.6. Determination of Critical Micelle Concentration (CMC)

A lipopeptide solution was prepared in Milli Q water, and the specific conductivity of each lipopeptide solution, in a concentration ranging from 0.06 to 18 mg/mL, was measured using a drop conductivity meter (HORIBA, Kyoto, Japan), at 25 °C. Conductivity values were plotted vs. lipopeptide concentration (mS × cm^−1^ vs. mg/mL), and the CMC was graphically determined [43].

### 3.7. Secondary Structure Determination by Circular Dichroism (CD)

Far-UV circular dichroism (CD) measurements were performed on a Jasco J-810 CD spectrometer (Tokyo, Japan) in a 0.1 cm path quartz cuvette (Hellma, Müllheim, Germany) and recorded after five runs. CD analyses were recorded in the presence of dipalmitoylphosphatidylglycerol (DPPG) and dipalmitoylphosphatidylcholine (DPPC) vesicles. For the preparation of small unilamellar vesicles, lipid dispersion in Milli-Q water was sonicated using a tip-sonicator (Vibra cell), until the solution became transparent. The final lipid concentration was 3 mM. Spectra were corrected for background scattering caused by the vesicles by subtracting the spectrum of a single vesicle solution from that of the peptides in the presence of vesicles [44]. Additional spectra were obtained in water and the presence of trifluoroethanol [50% TFE/H_2_O (*v*/*v*)]. The final peptide concentration was 0.2 mg/mL for all cases. CD spectra were deconvoluted by means of the CDPro software package (Colorado State University), using SELCOM 3 and CONTILL methods [45].

## 4. Conclusions

Here, we studied the effect of the substitution with non-proteinogenic D-amino acids and N-methyl amino acids on the therapeutic properties and enzymatic stability of two previously described compounds, namely TA4 peptide and C10:0-A2 lipopeptide. Both strategies were effective at increasing the therapeutic potential of the molecules.

The substitution of D-amino acids in both TA4 and C10:0-A2 at multiple sites in the peptide sequence reduced toxicity against human red blood cells and HeLa cells, possibly because a diminution in the experimental hydrophobicity of these molecules, as shown by the retention time in the RP-HPLC analysis, an important feature for hemolytic activity. However, these analogs showed lower antimicrobial activity, especially against gram (+) bacteria, than non-substituted ones. This observation could be explained by a decrease in the adoption of an amphipathic alpha-helix structure, as demonstrated by CD analysis in the presence of prokaryotic membrane-simulating mimetic environments (DPPG vesicles), where it was observed that multi-site D-amino acid substitution significantly decreased the adoption of secondary structure. This parameter is critical in activity against gram (+) bacteria.

A single or double substitution by N-methylated amino acids proved to be a highly effective strategy to decrease toxicity against the two eukaryotic systems tested, maintaining, and in some cases, increasing antimicrobial activity and resulting in highly selective compounds with high therapeutic indexes, especially against gram (+) and (−) bacteria. All N-Methyl substitution analogs presented significantly less experimental hydrophobicity, suggesting less interaction with the eukaryotic membranes, except C10:0-A2(9-NMeLys), which showed similar hydrophobicity and hemolytic activity than C10:0-A2. It is interesting to note that the more antimicrobial active lipopeptides were showed, the greater the contribution of the α-helix structure (C10:0-A2(9-NMeLys) and C10:0-A2(6-NMeLys), which reaffirms that the adoption of amphipatic α-helical structure is significant for these lipopeptides.

With respect to enzymatic stability, multiple D-substitution sites significantly increased enzymatic stability. On the other hand, single or double substitution by N-methylated amino acids was less favorable to enhance the enzymatic stability of the peptides and lipopeptides, in particular against digestive enzymes. This observation could be attributed mainly due to the numerous sites in the peptide sequences targeted by the enzymes tested.

TA4(dK), C10:0-A2(6-NMeLys), and C10:0-A2(9-NMeLys) were identified as the most promising molecules for further studies.

## Figures and Tables

**Table 1:** Sequence and physicochemical properties.

Identification	Sequence	Net Charge pH = 7	Experi Mental MM	rt	CMC (mM)
TA4	KLFKKLFKKLFK-NH_2_	+7	1567.16	17.2	ND
TA4(3,7-NMePhe)	KL(**NMeF**)KKL(**NMeF**)KKLFK-NH_2_	+7	1595.20	16.7	ND
TA4(dK)	**_d_K_d_K**LF**_d_K_d_K**LF**_d_K_d_K**LF**_d_K**-NH_2_	+7	1567.04	15.4	ND
C10:0-A2	C10:0-IKQVKKLFKK-NH_2_	+5	1413.08	17.8	5.74
C10:0-A2(5-NMeLys)	C10:0-IKQV(**NMe-K**)KLFKK-NH_2_	+5	1426.87	16.3	3.73
C10:0-A2(6-NMeLys)	C10:0-IKQVK(**NMe-K**)LFKK-NH_2_	+5	1426.99	16.1	3.63
C10:0-A2(9-NMeLys)	C10:0-IKQVKKLF(**NMe-K**)K-NH_2_	+5	1427.08	17.7	3.90
C10:0-A2(8-NMePhe)	C10:0-IKQVKKL(**NMe-F**)KK-NH_2_	+5	1427.03	15.3	2.01
C10:0-A2(dK)	C10:0-I**_d_K**QV**_d_K_d_K**LF**_d_K_d_K**-NH_2_	+5	1413.10	17.3	5.68

Experimental MM: correspond to ion [M]^+^, determined by Mass Spectrometry MALDI-TOF. rt: retention time determined by HPLC C18 analytical column for peptides and C4 column for lipopeptides. _d_K: D-Lysine; _d_L: D-Leucine; _d_F: D-Phenylalanine; NMe-K: N-methyl-L-Lysine; NMe-F: N-methyl-L-Phenylalanine; and C10:0: decanoic acid.

**Table 2:** MIC of synthesized compounds against Gram (+) and (−) bacteria and yeast.

Microorganism		TA4	TA4(3,7-NMePhe)	TA4(dK)	C10:0-A2	C10:0-A2(5-NMeLys)	C10:0-A2(6-NMeLys)	C10:0-A2(9-NMeLys)	C10:0-A2(8-NMePhe)	C10:0-A2(dK)
		MIC (µM)								
Gram negative	*E. coli* ATCC 35218	5.1	5.0	5.1	1.4	2.8	1.4	2.8	11.2	22.6
	*P. aeruginosa* ATCC 27853	2.6	1.2	10.2	1.4	5.6	0.7	0.7	5.6	1.6
Gram positive	*S. aureus* ATCC 25923	2.6	5.0	10.2	1.4	2.8	1.4	2.8	2.8	>90.6
	*S. aureus* RM * SA1	10.2	10.0	20.4	2.8	5.6	5.6	2.8	5.6	>90.6
	*E. faecalis* ATCC 29212	5.1	40.0	20.4	1.4	11.2	5.6	5.6	11.2	>90.6
Yeast	*C. albicans* PEEC 2	40.8	80.2	>163.3	90.6	179.5	44.9	89.7	179.5	90.6
	*C. tropicalis* DBFIQ 3	40.8	80.2	163.3	90.6	179.5	89.7	179.5	179.5	181.2

* Methicillin-resistant *Staphylococcus aureus*.

**Table 3:** Therapeutic Index of the synthesized compounds.

Identification	MIC Average (µM)			HC_50_ ± SD (µM)	IC_50_ ± SD (µM)	TI		
	Bacteria		Yeast			Bacteria		Yeast
	Gram (−)	Gram (+)				Gram (−)	Gram (+)	
TA4	3.9	6.0	40.8	>400	46.35 ± 10.13	102.6/11.9	66.7/7.7	9.8/1.1
TA4(3,7-NMePhe)	3.1	28.3	80.2	>400	86.56 ± 7.35	129.0/27.9	14.1/3.1	5.0/1.1
TA4(dK)	7.7	17.0	163.3	>400	>400	51.9	23.5	2.4
C10:0-A2	1.4	1.9	90.6	202.9 ± 17.5	32.62 ± 2.49	144.9/23.3	106.8/17.2	2.2/0.4
C10:0-A2(5-NMeLys)	4.2	6.5	179.5	>400	>400	95.2	61.5	2.2
C10:0-A2(6-NMeLys)	1.1	4.2	67.3	>400	399.90 ± 28.96	363.6	95.2	5.9
C10:0-A2(9-NMeLys)	1.8	3.7	134.6	298.5 ± 12.0	>400	165.8/222.2	80.7/108.1	2.2/3.0
C10:0-A2(8-NMePhe)	8.4	6.5	179.5	>400	>400	47.6	61.5	2.2
C10:0-A2(dK)	12.1	>90.6	135.9	>400	>400	33.1	<4.4	2.9

HC_50_ (µM) and IC_50_ values were determined by regression analyses of three replicate determinations. All values were expressed with a confidence of 95%.

## Data Availability

Not applicable.

## Supplementary Materials

The following supporting information can be downloaded at: https://www.mdpi.com/article/10.3390/antibiotics12050821/s1, Figure S1: Percentage of secondary structure types of peptides obtained by deconvolution of spectra by means of CDPro software package (Colorado State University), using SELCOM 3 and CONTILL methods. Figure S2: Percentage of secondary structure types lipopeptides obtained by deconvolution of spectra by means of CDPro software package (Colorado State University), using SELCOM 3 and CONTILL methods.
