# Supplementary material for: D- and N-Methyl Amino Acids for Modulating the Therapeutic Properties of Antimicrobial Peptides and Lipopeptides
Source: Antibiotics (Basel). 2023 Apr 27;12(5):821. doi: 10.3390/antibiotics12050821 (PMC10215143; doi:10.3390/antibiotics12050821)
Supplement: Supplementary file 1 [file antibiotics-12-00821-s001.zip › antibiotics-2360479-supplementary.pdf]

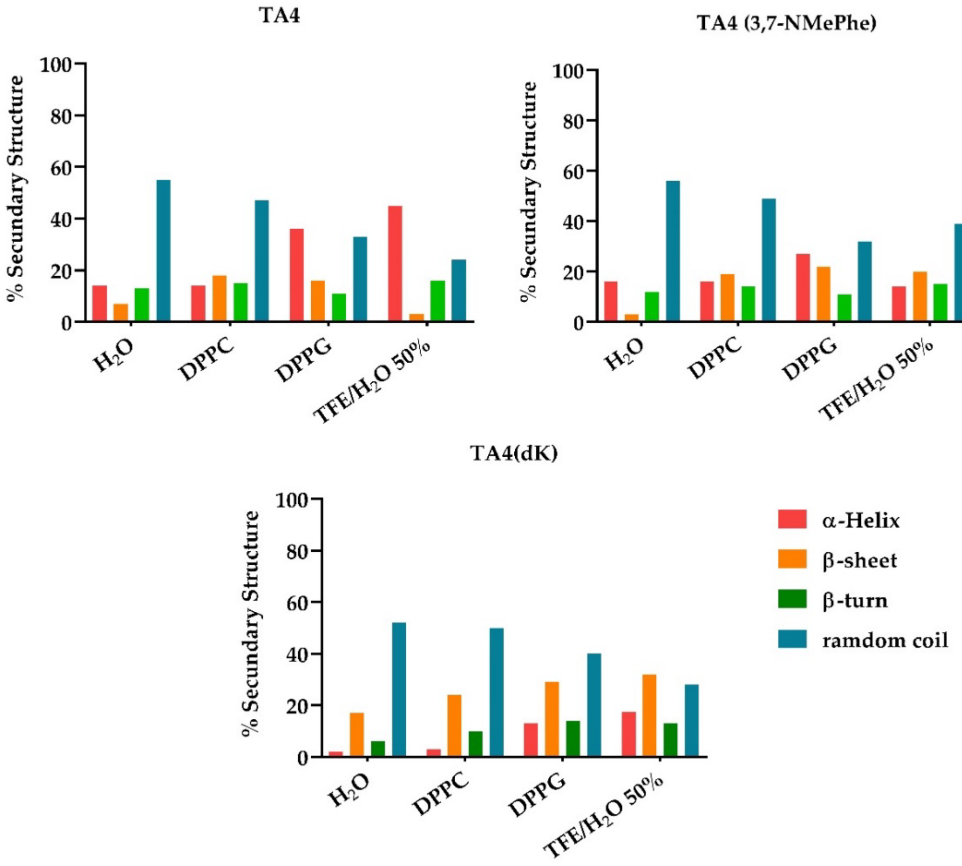

Figure 1S. Percentage of secondary structure types of peptides obtained by deconvolution of spectra by means of CDPro software package (Colorado State University), using SELCOM 3 and CONTILL methods.

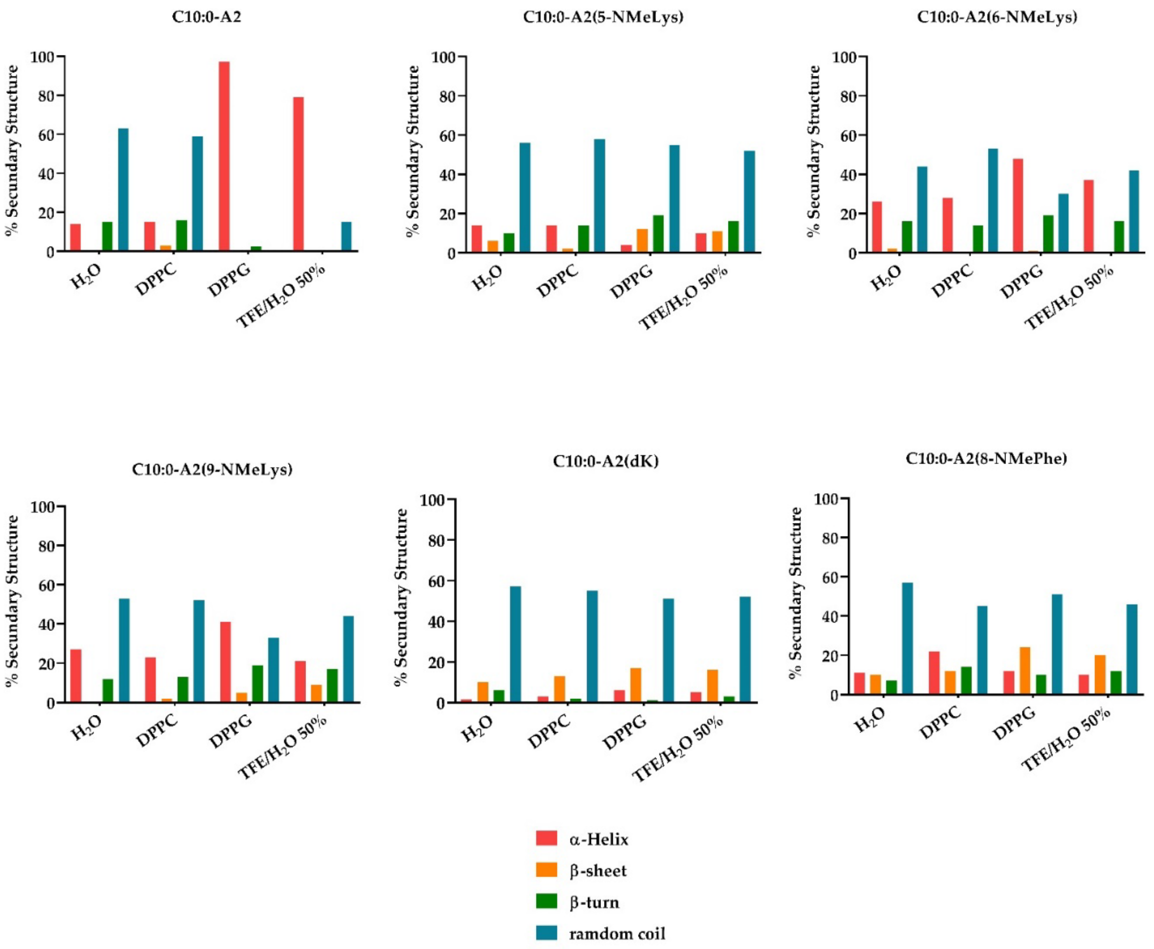

Figure 2S. Percentage of secondary structure types lipopeptides obtained by deconvolution of spectra by means of CDPro software package (Colorado State University), using SELCOM 3 and CONTILL methods
